# Amiodarone Induced Hyponatremia Masquerading as Syndrome of Inappropriate Antidiuretic Hormone Secretion by Anaplastic Carcinoma of Prostate

**DOI:** 10.1155/2014/136984

**Published:** 2014-04-08

**Authors:** Pinaki Dutta, Girish Parthan, Anuradha Aggarwal, Santosh Kumar, Nandita Kakkar, Anil Bhansali, Fabio Rotondo, Kalman Kovacs

**Affiliations:** ^1^Department of Endocrinology, Post Graduate Institute of Medical Education and Research, Nehru Hospital, 4th Floor, F Block, Chandigarh 160012, India; ^2^Department of Urology, Post Graduate Institute of Medical Education and Research (PGIMER), Chandigarh 160012, India; ^3^Department of Histopathology, Post Graduate Institute of Medical Education and Research, Chandigarh 160012, India; ^4^Department of Laboratory Medicine and Surgical Pathology, St. Michael's Hospital, Room 2-101V, 30 Bond Street, Toronto, ON, Canada M5B 1W8

## Abstract

Syndrome of inappropriate antidiuretic hormone secretion (SIADH) is one of the most common causes of hyponatremia. The usual causes are malignancies, central nervous system, pulmonary disorders, and drugs. Amiodarone is a broad spectrum antiarrhythmic agent widely used in the management of arrhythmias. The different side effects include thyroid dysfunction, visual disturbances, pulmonary infiltrates, ataxia, cardiac conduction abnormalities, drug interactions, corneal microdeposits, skin rashes, and gastrointestinal disturbances. SIADH is a rare but lethal side effect of amiodarone. We describe a 62-year-old male who was suffering from advanced prostatic malignancy, taking amiodarone for underlying heart disease. He developed SIADH which was initially thought to be paraneoplastic in etiology, but later histopathology refuted that. This case emphasizes the importance of detailed drug history and the role of immunohistochemistry in establishing the diagnosis and management of hyponatremia due to SIADH.

## 1. Introduction

Syndrome of inappropriate antidiuretic hormone secretion (SIADH) is one of the most common causes of hyponatremia. This syndrome is caused by various malignancies, lung diseases, central nervous system disorders, and drugs. The commonest malignancy associated with SIADH is small cell carcinoma of the lung [1]. There are a few case reports of SIADH in patients with small cell and anaplastic carcinoma of prostate [2]. Amongst various drugs selective serotonin reuptake inhibitors, carbamazepine, thiazide diuretics, chlorpropamide, and alkylating agents are commonly associated with SIADH [3–6]. Amiodarone is extensively used in clinical practice for a wide variety of arrhythmias. The adverse effect profile is very broad; however, there are very few cases of hyponatremia due to SIADH reported with this drug [7–16]. We describe one such case in an elderly male which was initially thought to be due to ectopic secretion of vasopressin by an anaplastic carcinoma of prostate but later refuted by immunohistochemistry of the tumor and normalization of serum sodium level following omission of amiodarone.

## 2. Case Report

**Figure 1:**
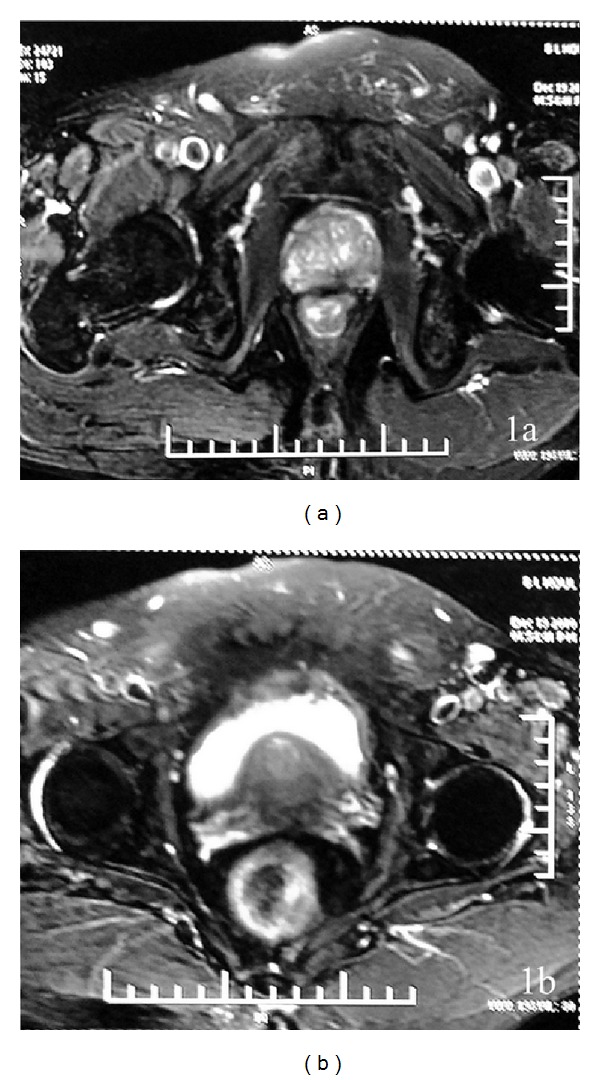
MRI showed ill-defined low intensity signal in peripheral zone of prostate and adjacent portion of bilateral seminal vesicles.

**Figure 2:**
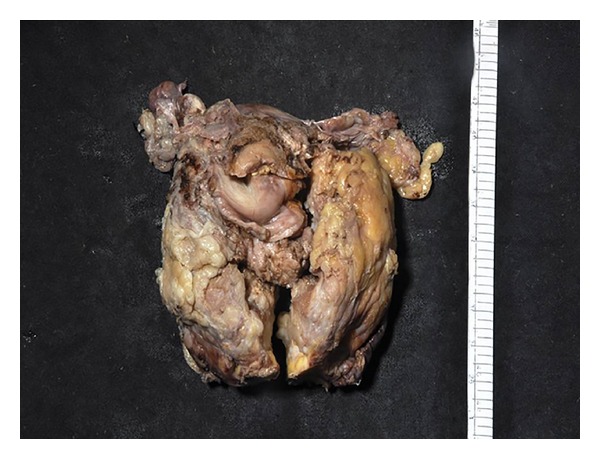
Resected prostatectomy specimen showing large prostatic mass with variegated appearance with infiltration of capsule and bilateral seminal vesicles.

A 63-year-old male, known to be hypothyroid for the past three years, on treatment with 100 *μ*g of L-thyroxine presented to his primary care physician with history of 3 episodes of syncopal attacks 8 months back. He was a smoker with history suggestive of chronic bronchitis. He was also hypertensive for the last 4 years. His blood pressure was stable on 100 mg of losartan per day. Electrocardiogram and 24-hour holter revealed runs of ill sustained ventricular tachycardia and he was started on 100 mg of amiodarone per day 8 months back. He had history of prostatism since 2008 and was on tamsulosin 0.4 mg once daily. He had some relief, but in 2010 there was recurrence of symptoms. His ultrasound revealed enlarged prostate of size 5.2 × 4.8 × 5.4 cm with a residual urine volume of 75 mL. The serum prostate specific antigen (PSA) was found to be elevated (152 ng/mL, normal <5). MRI of prostate reconfirmed the same and showed ill-defined low intensity signal in peripheral zone of prostate and adjacent portion of bilateral seminal vesicles suggestive of stage III disease (Figures 1(a) and 1(b)). Biopsy showed adenocarcinoma of the prostate (Gleason's Grade 3 + 3). Bone scan was normal and he had normal serum electrolyte at that time. The patient was started on goserelin acetate (Zoladex 10.8 mg) once every three monthly as he refused orchidectomy. The PSA level decreased to 6 ng/mL in mid-2012. In May 2013, he was hospitalized elsewhere with acute urinary retention requiring catheterization followed by hematuria. His sonogram of the prostate showed the same findings and PSA was 23.16 ng/mL at this juncture. He was subjected to bilateral orchidectomy, cystoscopy, and transurethral prostate resection (Figure 2). Two months later he presented with generalized tiredness and disorientation. His hemoglobin was 8.8 gm/dL, urea 20 mg/dL, creatinine 0.8 mg/dL, sodium 124 mmol/L, potassium 3.9 mmol/L, uric acid 2.2 mg/dL, protein 5.4 g/dL, and serum albumin 3.6 g/dL. His liver function test was normal. He was started on fluid restriction, 10–15 gms of added salt per day, and tolvaptan 60 mg per day without much improvement in his symptoms. This time he was admitted to our hospital for further evaluation and treatment. On evaluation, his hemoglobin was 8.6 mg/dL, and he had normal renal function, sodium 109 mmol/L, serum cortisol 738 nmol/L (*N*: 350–540), T_3_ 1.2 ng/mL (*N*: 0.8–2.0), T_4_ 10.2 *μ*g/dL (*N*: 4.8–12.7), TSH 4.64 mIU/mL (*N*: 0.27–4.2), plasma osmolality 242 mOsm/kg (*N*: 275–295), urine osmolality 504 mOsm/kg (*N*: 50–1200), and spot urine sodium 105 mmol/L (*N* > 20). Follow-up bone scan revealed increased osteoblastic activity in right fifth rib suggestive of metastatic process. Fluorodeoxyglucose-positron emission tomography (FDG PET) showed avid lesions in the prostate, pelvic bones, abdominal lymph nodes, left transverse abdominal muscles, and liver. Tolvaptan was continued and patient was advised liberal oral salt (15 gms/day) and to restrict his fluid intake. Hyponatremia persisted despite these measures. It was thought to be because of SIADH due to ectopically secreted vasopressin from prostatic malignancy, which was negated by immunohistochemistry. A literature review at this juncture made us suspect the possibility of amiodarone induced hyponatremia. Amiodarone was stopped and on the third day of the discontinuation of the drug, serum sodium improved to 122 mmol/L. Metoprolol was introduced replacing amiodarone; however, patient succumbed to refractory ventricular tachycardia in the next five days. Autopsy could not be performed.

**Figure 3:**
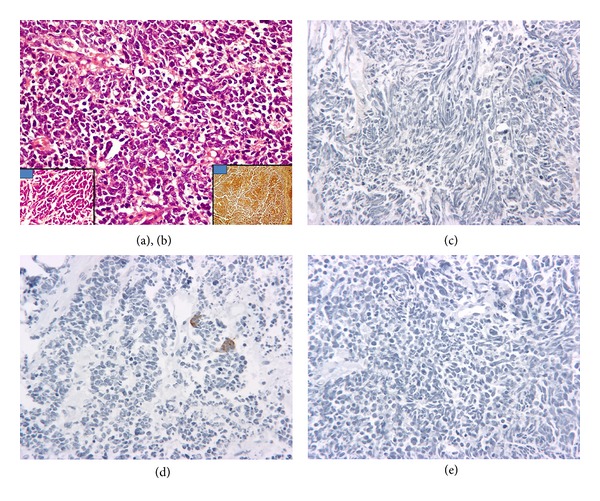
((a) and (b)) Microphotograph from the prostate shows a tumor present in sheets with hyperchromatic nuclei and scanty cytoplasm consistent with morphology of small cell carcinoma H&E ×200. Insets show (a) tumor cells at high power which show moulding H&E ×400 and (b) strong positivity with neurone specific enolase IHC ×100. (c) Immunohistochemistry showing vasopressin negativity. (d) Immunohistochemistry for the carrier protein neurophysin is negative. (e) Immunohistochemistry for prostate specific antigen (PSA) is conspicuously negative suggestive of dedifferentiated tumor.

**Figure 4:**
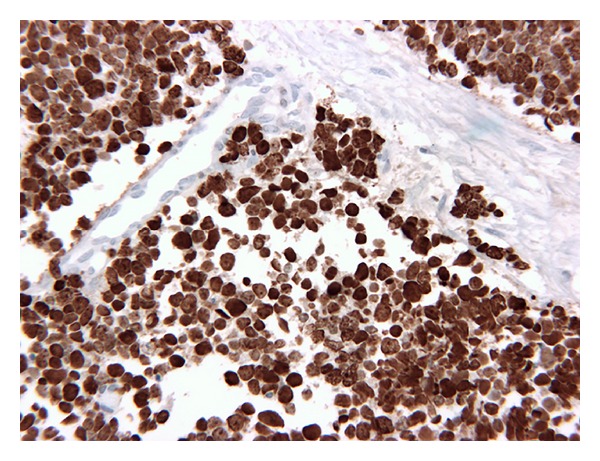
Immunohistochemistry showing strong positivity for Ki-67, a marker for proliferation.

Light microscopy of the biopsy specimen at our institute showed nontumorous prostate containing glands, muscle, and connective tissue. Adjacent to it there was a cellular neoplasm. The tumor exhibited a diffuse pattern arranged predominantly in sheets, nests, and trabeculae and is composed of relatively small pleomorphic cells possessing a narrow rim of mildly acidophilic cytoplasm, elongated or rounded hyperchromatic nuclei, and inconspicuous nucleoli. Apoptotic nuclei and mitotic figures (6–8/10 hpf) are easily recognized. The tumor was seen infiltrating the muscle. Numerous bizarre tumor cells are also seen. In addition 2% of tumor cells showed back to back arrangement in glandular configuration. For immunohistochemistry, the streptavidin-biotin-peroxidase complex method was used. The tumor cells were positive for CD-56 and neuron specific enolase (Figures 3(a) and 3(b)) and conclusively negative for vasopressin and neurophysin (the carrier protein of vasopressin, Figures 3(c) and 3(d)). No immunopositivity was noted in the tumor cells for prostate specific antigen (PSA, Figure 3(e)). Immunostaining performed for Ki-67 using MIB-1 antibody showed abundant nuclear positivity indicating a rapid cell proliferation rate (Figure 4). Additionally, the tumor cells were negative for LCA, TTF-1, and chromogranin. These findings were suggestive of dedifferentiation of small cell carcinoma of patients towards an anaplastic carcinoma.

## 3. Discussion

SIADH was first reported by Schwartz et al. in 1957 in patients with bronchogenic carcinoma and meningitis [1]. The essential criteria include normovolemic hyponatremia in a patient who is euthyroid and eucortisolic [5]. In 1971 four cases of chlorpropamide-induced hyponatremia were described by Weissman et al. [5]. Since then, a variety of drugs have been added to the list of drugs that cause SIADH.

The clinical symptoms of SIADH are nonspecific and are essentially those of hyponatremia. The magnitude and rate of development of hyponatremia determines the presence and severity of symptoms. In general, slowly progressive hyponatremia is associated with fewer symptoms. Patients with moderate chronic hyponatremia may have decreased reaction time, cognitive slowing, and ataxia resulting in frequent falls. Sudden significant decline in serum sodium may lead to altered sensorium and seizures. The symptoms pertaining to the etiology of SIADH may be present if the underlying cause is CNS or pulmonary disorders.

Amiodarone, a benzofuran derivative, was developed by a Belgian company (Labaz) in 1961 by Tondeur and Binon as an antianginal drug. Since then it has been used extensively as a broad spectrum antiarrhythmic agent [17]. The first case of amiodarone-induced hyponatremia was reported in 1996. To the best of our knowledge a total of 11 cases of amiodarone-induced SIADH have been published in English literature [7–16]. The summary of reported cases is shown in Table 1.

Amongst the 11 reported cases, the majority (9/11) were elderly men. Elderly subjects as such appear to be particularly at risk for drug-induced SIADH. In a study of 736 cases of selective serotonin reuptake inhibitor-induced SIADH, 75% of patients were over 65 years of age [4–6].

The time from the introduction of amiodarone to the development of SIADH varied from 3 days to 6 months in the reported cases. The hyponatremia usually occurred within the first 2 weeks in those who received loading doses of amiodarone. In contrast, it occurred at 2 weeks to 6 months in patients receiving maintenance doses. The level of serum sodium varied from 105 to 120 (mean = 115) mmol/L.

In 7 cases, the serum sodium level normalized within 7–14 days after discontinuation of amiodarone along with fluid restriction. In our case the serum sodium started improving within 3 days of stoppage of the drug. Amiodarone-induced SIADH seems to be more prevalent after a loading dose of the drug. However, because of the long half-life of amiodarone, the cumulative effect on the development of SIADH remains undefined. Amiodarone-induced SIADH is a rare adverse effect, which occurs predominantly in elderly patients, who typically have multiple comorbidities, especially cardiovascular disease.

The only definitive treatment of SIADH is the elimination of its underlying cause. In all but 3 cases, amiodarone was discontinued and fluid restriction was instituted. In the remaining cases, the dose was decreased. One patient required hemodialysis due to low serum sodium accompanied by development of altered mental status.

The mechanism of SIADH secondary to amiodarone and why it occurs in a limited number of cases exposed to the drug are unclear. It could possibly be due to the independent secretion of arginine vasopressin relative to plasma osmolality or inherited mutations of the aquaretic (i.e., water channel regulating) vasopressin receptor. Most medications cause SIADH either by sensitizing the kidneys to antidiuretic hormone, by stimulating the release of antidiuretic hormone, or by both. Of note Shavit and Sherer speculated that amiodarone might induce SIADH by its channel-modulating properties on either renal or neural tissues [13]. It is definitely not due to thyroid dysfunction as none of the reported cases were hypothyroid.

The severity of hyponatremia directly correlates with mortality in hospitalized patients [3, 18]. Therefore symptomatic treatment as well as removal of the cause, if possible, is required. In the index case, we continued tolvaptan, restricted fluid, and gave liberal salt. With these measures the hyponatremia improved as happened in previously reported cases [19].

In the index case initially the SIADH was suspected to be due to ectopic secretion of vasopressin by the advanced prostatic malignancy as there are instances of it in the literature [2]. However negativity of the histopathology specimen for vasopressin and neurophysin is against it. There is a remote possibility that vasopressin was released from and not stored in the tumor cells. This suggestion is unlikely because immune-positivity is practically always seen in tumors producing excessive amount of hormones ectopically. As our patient was euthyroid and eucortisolic without any underlying kidney disease, amiodarone treatment appeared to be the cause of SIADH. This study provides substantial evidence that amiodarone was responsible for SIADH. This was confirmed by improvement in patient's hyponatremia within 3 days of discontinuation of the drug.

In conclusion, this is a case of SIADH due to an iatrogenic reason. Prostate cancer was erroneously thought to be the cause. Amiodarone induced SIADH can occur as early as 3 days after receiving a loading dose or as late as 6 months in patients on maintenance doses. Elderly males appear to be more predisposed. High index of suspicion is required to suspect this rare but life threatening adverse event when treating patients with amiodarone. The severity of hyponatremia directly correlates with increased mortality.

## Figures and Tables

**Table 1:** Summary of cases of SIADH Induced by amiodarone, including the present case.

Serial number	Author and year	Age	Gender	Sodium level at diagnosis (mmol/L)	Amiodarone dose and duration	Time to develop hyponatremia	Management	Day on which serum sodium normalized
1	Ruiz et al. 1996 [8]	67	F	110	Not mentioned	4 Months	Discontinuation	Unknown
2	Odeh et al. 1999 [7]	62	F	120	300 mg qd	6 Months	Discontinuation	5 Days
3	Patel and Kasiar 2002 [9]	67	M	117	200 mg qd	3 Months	Fluid restriction, Discontinuation of therapy	3 Days
4	Ikegami et al. 2002 [11]	63	M	119	800 mg qd	7 Days	Amiodarone dose reduced to 100 mg qd, fluid restriction	28 days
5	Ikegami et al. 2002 [11]	87	M	121	200 mg qd for seven days then 100 mg qd	15 Days	Fluid restriction only	14 Days
6	Aslam et al. 2004 [10]	72	M	117	2 g qd (loading dose)	5 Days	Decrease dose to 200 mg qd	Normalized on 2nd day, Stable on 14th day
7	Paydas et al. 2008 [12]	58	M	107	Not mentioned	5 Months	Cessation of treatment	14 Days
8	Shavit and sherer 2007 [13]	85	M	122	Not mentioned	30 Days	Cessation of treatment	A few days, stable at 3rd month
9	Singla et al. 2013 [14]	66	M	120	Loading followed by 400 mg four times a day	3 Days	Discontinuation of amiodarone and hemodialysis	Not Mentioned
10	Afshinnia et al. 2011 [15]	72	M	117	150 mg IV loading, 900 mg over 24 hours, 400 mg TDS	7 days		16 Days
11	Pham et al. 2013 [16]	84	M	105	Loading followed by 400 mg 8 hourly ×3 days. Then 400 mg OD	11 Days	Hypertonic saline, Fluid restriction, Demichlocycline,	10 Days
12	Dutta et al. (Present Case)	62	M	109	100 mg OD	9 Months	Fluid restriction, stoppage of amiodarone	3 Days
