# Corrigendum to “Amiodarone Induced Hyponatremia Masquerading as Syndrome of Inappropriate Antidiuretic Hormone Secretion by Anaplastic Carcinoma of Prostate”

**DOI:** 10.1155/2018/1604016

**Published:** 2018-10-18

**Authors:** Pinaki Dutta, Girish Parthan, Anuradha Aggarwal, Santosh Kumar, Nandita Kakkar, Anil Bhansali, Fabio Rotondo, Kalman Kovacs

**Affiliations:** ^1^Department of Endocrinology, Post Graduate Institute of Medical Education and Research, Nehru Hospital, 4th Floor, F Block, Chandigarh 160012, India; ^2^Department of Urology, Post Graduate Institute of Medical Education and Research (PGIMER), Chandigarh 160012, India; ^3^Department of Histopathology, Post Graduate Institute of Medical Education and Research, Chandigarh 160012, India; ^4^Department of Laboratory Medicine and Surgical Pathology, St. Michael's Hospital, Room 2-101V, 30 Bond Street, Toronto, ON, Canada M5B 1W8

In the article titled “Amiodarone Induced Hyponatremia Masquerading as Syndrome of Inappropriate Antidiuretic Hormone Secretion by Anaplastic Carcinoma of Prostate” [1], the discussion section was similar to that of Pham et al. [2], cited in the article as reference 16. The authors apologize for the text reuse from this article. Also, additional references should have been cited, included in the text below as references 20-23 [3–6]. The Discussion should read as follows:

## Discussion

SIADH was first reported by Schwartz et al. in two patients, one with bronchogenic carcinoma and another with cerebromalacia [1]. Later, the diagnostic criteria for SIADH were propounded by Barrter and Schwartz, which includes hypoosmolar hyponatremia, inappropriately concentrated urine (in the presence of hypo-osmolality), clinical euvolemia, elevated urinary sodium, normal thyroid and adrenal functions, and absence of diuretics [20].

Common causes of SIADH include various malignancies (e.g., lung, pancreas, prostate, and duodenum), infections of lung and central nervous system, intracranial mass lesions [21] and hemorrhage, and idiopathic. A wide variety of drugs are associated with SIADH, including chlorpropamide, clofibrate, carbamazepine, vincristine, selective serotonin reuptake inhibitors, tricyclic anti-depressants, and NSAIDs [3].

Amiodarone is a class III anti-arrhythmic agent, which is extensively used for a wide variety of arrhythmias including supraventricular and ventricular arrhythmias. Amiodarone has several well-known adverse effects, like pulmonary fibrosis, hepatotoxicity, thyroid dysfunction (both hypo- and hyperthyroidism), and corneal deposits. Hyponatremia is a rare side effect of amiodarone and, to the best of our knowledge, a total of 11 cases of amiodarone-induced SIADH have been published in the English literature [5-15].

Among the 11 reported cases of amiodarone-induced SIADH, 9 were men, 8 above 60 years, and one aged 58 years. There were only two females and both were > 60 years of age. None of the reported patients had any underlying malignancy. The time for the development of SIADH after initiation of amiodarone varied from 3 days to 6 months. SIADH tended to develop earlier in those who received a loading dose of amiodarone (within first 2 weeks) as compared to those who received only the maintenance doses (2 weeks to 6 months). Our patient developed SIADH after 9 months of amiodarone therapy and this is the longest lag period reported in the literature. Serum sodium levels in published patients ranged from 105 to 120mEq/L, with mean sodium being 115 mEq/L. Serum sodium normalized rapidly after discontinuation of amiodarone/reduction of dosage along with fluid restriction in majority of the patients, within 5 days in 4 patients and within 2 weeks in 3 patients, while in 2 patients serum sodium normalized after 16 and 28 days. The time required for the normalization of serum sodium was not known in two patients. Our patient's serum sodium started to improve within 3 days of discontinuation of amiodarone. The rapid reversal of SIADH after discontinuation of amiodarone is perplexing, given the long half-life of the drug.

The mechanism of amiodarone-induced SIADH is unknown. It is also unclear why only a few patients were exposed to amiodarone develop SIADH. It could be due to release of antidiuretic hormone or by a direct effect of amiodarone on vasopressin (V2) or aquaporin receptors in kidneys [13]. The long tissue half-life but rapid recovery of hyponatremia in few cases and occurrence only in small subset of patients exposed is against the direct involvement by the drug. Amiodarone-induced hypothyroidism could be a remote possibility but none of the reported cases had it and our patient was euthyroid on replacement. It was also proposed that amiodarone induces SIADH by modulation of channels in neurons or kidney [13].

The management of SIADH includes fluid restriction, hypertonic saline, oral salt, loop diuretics, urea, lithium, demeclocycline, and, most importantly, management of underlying cause. Recently vasopressin receptor antagonists were approved for the management of SIADH [22]. In the 11 published cases, the drug was discontinued in seven patients, while dose was reduced in three. Fluid restriction was employed in four patients; one patient was treated with demeclocycline and one patient required hemodialysis. In our patient, fluid restriction, liberal salt intake, and tolvaptan were employed along with discontinuation of amiodarone and the patient's serum sodium improved rapidly. This report is the first instance of tolvaptan use in amiodarone-induced hyponatremia.

In the index patient, SIADH was initially suspected to be due to ectopic secretion of vasopressin by the prostatic malignancy, as previously reported in the literature [2, 23]. However, negative immunohistochemistry for vasopressin and neurophysin in the tumor tissue refutes this possibility. There is a remote possibility that vasopressin was released from the tumor tissue and not stored within. This suggestion is unlikely because immunopositivity is practically always seen in tumors producing hormones ectopically. Our patient had fulfilled the criteria for SIADH and lack of improvement of serum sodium with fluid restriction and tolvaptan, and prompt improvement of hyponatremia after discontinuation of amiodarone strongly suggests that amiodarone was the cause of SIADH in our patient.

In conclusion, we report a case of SIADH in an elderly male with anaplastic carcinoma of the prostate, in which prostatic malignancy was erroneously thought to be the cause but turned out to be induced by amiodarone. SIADH is a rare side effect of amiodarone and high index of suspicion is required to suspect this life threatening adverse event.
